# Cardiac Implantable Electronic Device Infections—Decision-Making Process in Complex Patients: Report of 3 Cases

**DOI:** 10.1177/2324709619831320

**Published:** 2019-02-21

**Authors:** Assad Mohammedzein, Aaisha Mozumder, Scott Milton

**Affiliations:** 1 Texas Tech University Health Science Center, Amarillo, TX, USA

**Keywords:** cardiac implantable electronic devices, endocarditis, lead infection, pocket infection

## Abstract

Cardiac implantable electronic devices infections are becoming increasingly frequent with more of these devices being implanted in elderly patients with multiple comorbidities. They carry high morbidity and mortality if not treated promptly, which often entails removal of the entire system. Early recognition is paramount, and a multidisciplinary team is required. In this case report, we go through unique and challenging presentations of such patients with cardiac implantable electronic devices–related issues who presented to our institution, and we describe individual approaches to management and review the literature.

## Background

Cardiac implantable electronic devices (CIEDs) infections are relatively common with an incidence ranging between 0.8% and 5.7%^1^; they carry higher morbidity and mortality in comparison to those patients who present with other device-related complications. Coagulase negative *Staphylococcus* and *Staphylococcus aureus* are the most common pathogens isolated,^2^ and use of CIEDs has probably contributed to the shift in prevalence of these organisms as a cause of infective endocarditis. CIED infections are generally divided into 2 groups: those confined to the “pocket” and systemic. These categories are not mutually exclusive, and the 2 forms may coexist. Pocket infection refers to infection involving the subcutaneous pocket containing the pulse generator and the subcutaneous part of the leads.

In this cases-based discussion, we go through different presentations of cardiac device–related infections and highlight important and challenging aspects of management.

## Case Presentation

### Case 1

A 42-year-old male with a past medical history of chronic systolic heart failure (ejection fraction [EF] 25%), status post implantable cardioverter-defibrillator (ICD) 2 years ago, diabetes mellitus, and hypertension, presented to the emergency department complaining of fever for 1 day and intermittent purulent discharge from the ICD site for the past 2 months. He has a history of medication noncompliance and current illicit drug use.

Evaluation revealed temperature of 38.6°C, heart rate 112 beats per minute, blood pressure 99/55 mm Hg, respiratory rate 24 breaths per minute, and O_2_ saturation 94% breathing ambient air. There was erythema and swelling around the ICD pulse generator. Closed sinus was seen with no active discharge, and the area was warm and tender to palpation.

Blood cultures were collected, and he was started on vancomycin and meropenem due to his penicillin allergy. His blood pressure dropped further, and he went into septic shock with respiratory failure requiring intubation. Blood culture grew methicillin-sensitive *Staphylococcus aureus* (MSSA) in 2 sets.

Transthoracic echocardiogram (TTE) showed EF 20%, with no evidence of lead or valve vegetations. Antibiotic was de-escalated to cefazolin, and he was extubated successfully in 2 days. The ICD pulse generator and lead were extracted successfully without complication. Culture from the pocket grew *MSSA*, but blood culture remained negative. He was fitted with a LifeVest and had finished 6 weeks of cefazolin intravenously with no recurrence of infection 5 months after discharge. He was not considered for new device implant unless he proves commitment to quitting drug use.

### Case 2

An 81-year-old male with history of heart transplant 30 years ago on chronic immunosuppressive therapy and a permanent pacemaker implanted at the time of transplant with 3 revisions most recently 7 years ago. He has been treated for recurrent *S epidermidis* bacteremia for several years with no clear source.

The patient presented this time with 1 week of fever and malaise. The pacemaker site looked noninfected. Blood culture grew methicillin-resistant *S epidermidis* in 2 sets. Thorough workup including TEE was negative for a source. Since he had high-grade bacteremia (2 sets of positive blood culture) with an organism commonly implicated in device infection, a decision was made to treat as device endocarditis. The pacemaker pulse generator and leads were extracted with the use of laser. There was no evidence of vegetations on the tricuspid valve or leads on intracardiac echocardiogram, which was used to guide the procedure and monitor for complications. Culture of a cloudy fluid from the lumen of the atrial lead showed heavy growth of coagulase negative *Staphylococcus*, further supporting the diagnosis of device endocarditis.

He required temporary transvenous pacing for few days and discharged to a nursing facility to continue vancomycin for a total of 4 to 6 weeks. At 3-month follow-up, he had no recurrence of sepsis; neither did he require device reimplantation.

### Case 3

A 54-year-old male with a past medical history of nonischemic cardiomyopathy, EF 20% and ICD in situ, gout with extensive tophi, chronic lymphocytic leukemia on ibrutinib, and venous thromboembolism on rivaroxaban. He felt as if the ICD fired. Vitals were normal and ICD site looked normal. There was no evidence of arrhythmia or shock therapy on device interrogation.

During hospital stay, he became febrile and hypotensive, blood cultures were collected, and he was started on broad-spectrum antibiotics. Blood cultures grew MSSA in 2 sets. Antibiotics were de-escalated to cefazolin. Transesophageal echocardiogram (TEE) was performed with no evidence of vegetations. He also noted drainage of white cheesy material from an elbow tophus; cultures from these grew MSSA as well. Blood cultures remained negative when repeated 2 days after starting the antibiotic.

The infected tophus was determined to be the likely source of bacteremia, and with the clearance of bacteremia and absent vegetation, the ICD was left in place. He was discharged to a skilled nursing facility to complete 2 weeks of intravenous antibiotics. There was no evidence of recurrence of infection at 2-month follow-up.

## Discussion

Cardiac device infections are increasing in frequency as more of these devices are being implanted. Risk factors include immunosuppression therapy, chronic illness including diabetes mellitus and chronic kidney disease, and older age. The type of device and use of prophylactic antibiotic also play a role, with higher incidence in ICDs compared with pacemakers.

**Figure 1.**
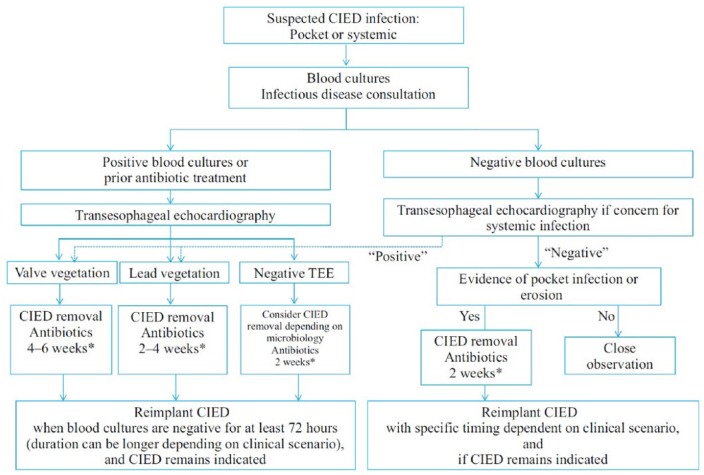
Management of suspected CIED (cardiac implantable electronic device) infection. *Antimicrobial therapy should be at least 4 to 6 weeks for endocarditis (4 weeks for native valve, 6 weeks for prosthetic valve or staphylococcal valvular endocarditis). If lead vegetation is present in the absence of a valve vegetation, 4 weeks of antibiotics for *Staphylococcus aureus* and 2 weeks for other pathogens is recommended.^6^

The first case illustrates a straightforward presentation with clear infection involving the pocket. Since the patient is bacteremic, it indicates the intravenous leads are invariably involved even if no vegetations are seen on TEE. Ideally, all patients suspected of having CIED infection should get TEE as it is more sensitive in detecting lead and valve vegetations, 90% compared with only 40% in TTE.^3^ TEE also helps diagnose valve endocarditis and paravalvular abscess, both determining the duration of antibiotic (Figure 1). Fluorodeoxyglucose (^18^F-FDG) positron emission tomography/computed tomography scanning is particularly helpful in situations where pocket infection is suspected but the patient is not showing signs of local or systemic infection, and it has good sensitivity and specificity for pocket infection but not endocarditis. Patients with pocket infection and bacteremia should be treated as infective endocarditis with extraction of the entire device, often with the use of laser, as the leads can become incorporated in the myocardial endothelium within a few weeks. Vegetations greater than 1 cm should include cardiothoracic surgical consultation for removal, as the risk of embolization is much higher. Studies demonstrated higher recurrence rate and mortality with incomplete removal of the device.^4,5^ Intravenous antibiotics are warranted and should be bactericidal against the organism and treated usually for 6 weeks, from the day of device removal.

If needed, the new device should be implanted in a different place, once the infection is controlled and blood culture remain negative for at least 72 hours (the same applies if the infection was involving the pocket only, with negative blood culture and no vegetations). On the other hand, if there is valve vegetation, reimplantation should be delayed for 2 weeks. External pacing is an option in the interim. Our patient is particularly challenging as his drug abuse and noncompliance puts him at high risk for reinfection. A new device has not been offered to him by his cardiologist.

Case 2 is different, as there was no clinical evidence of device infection with no vegetations on TEE. Complete workup is indicated in this situation to rule out other sources of infection. Our patient continued to have *S epidermidis* bacteremia, a common organism implicated in up to 60% of CIED infections,^2^ despite long courses of intravenous antibiotic, indicating a persistence source. He had no other implanted hardwire. In this case, treating as infective endocarditis is reasonable.

Although he required a brief period of temporary pacing, he did well afterward with negative blood cultures and did not require another permanent pacemaker. Around 20% to 30% of these patients do not require device reimplantation.^7^

If the isolated organism is not one of the typical causes of CIED, for example, *Streptococcus pneumoniae* or *Escherichia coli*, a trial of antibiotic therapy is appropriate with reevaluation for any recurrence of infection.

The third patient was challenging because the source was not apparent initially. Later, he was found to have infected tophus in the right elbow with the same organism isolated in blood culture. It is important to perform TTE in such cases to make sure there are no vegetations, as seeding of the device can occur from bacteremia. The patient was discharged on a 2-week course of antibiotics. Repeat blood culture after finishing antibiotics remained negative.

The 3 cases illustrate how variable the clinical presentations of device infection can be. It also shows that medical management alone is not enough as demonstrated clearly in the second case, which despite the use of several courses of appropriate antibiotic continued to develop recurrent bacteremia, and an earlier referral should have been thought in this case. These patients were all immunocompromised and at particularly high risk of infection; the first is a methamphetamine user and diabetic, the second is on immunosuppressive therapy for heart transplant, and the third with leukemia on chemotherapy, and we suggest being extra vigilant in such a group of patients. We feel those real-world cases will help front field physicians, who will invariably come across such patients, to maintain a low threshold and promptly make the decision of referral to a specialist in the field even if the source of infection is not apparent or the device/leads looked normal.

## Conclusion

Modern medicine has changed the epidemiology of endocarditis. Previously, *Streptococcus viridans* was the most common pathogen isolated. Now *Staphylococcus* species are far more common, likely from the use of either permanent or temporary implantable devices. The field of cardiac devices has expanded significantly in the last quarter century, as well as the rate of associated complications, particularly infections. As seen from these scenarios, treatment needs to be tailored individually. A multidisciplinary approach involving infectious disease consults and specialists in CIED infections and lead extraction is recommended. Furthermore, a high level of suspicion toward the indwelling device is warranted. Unfortunately for many patients, curing of the infection can only occur with extraction of the entire device, followed by antibiotics bactericidal toward the isolated pathogen. Prevention is key but can be difficult in patients with risk factors similar to our patients described here.
